# Prognostic factors for olfactory groove meningioma with nasal cavity extension

**DOI:** 10.18632/oncotarget.23461

**Published:** 2017-12-19

**Authors:** Ji Zhang, Ke Sai, Zheng-Quan Zhu, Fu Hua Lin, Zi-Feng Wang, Yong-Ming Chen, Chun-Yu Huang, Yun-Lin Ye, Xiao-Li Wang, You-Ping Li, Shu-Xin Sun, Wei-Ying Zhong, Jian-Bin Chen, Yun-Qiang Yang

**Affiliations:** ^1^ Department of Neurosurgery, State Key Laboratory of Oncology in South China, Sun Yat-sen University Cancer Center, Collaborative Innovation Center for Cancer Medicine, Guangzhou, China; ^2^ Department of Neurosurgery, Tumor Hospital Affiliated of Xinjiang Medical University, Ürümqi, China; ^3^ State Key Laboratory of Oncology in South China, The Cancer Center of Sun Yat-sen University, Collaborative Innovation Center of Oncology, Guangzhou, China; ^4^ Department of Gastric Surgery, State Key Laboratory of Oncology in South China, Sun Yat-sen University Cancer Center, Collaborative Innovation Center for Cancer Medicine, Guangzhou, China; ^5^ Department of Endoscopy, State Key Laboratory of Oncology in South China, Sun Yat-sen University Cancer Center, Collaborative Innovation Center for Cancer Medicine, Guangzhou, China; ^6^ Department of Urology, State Key Laboratory of Oncology in South China, Sun Yat-sen University Cancer Center, Collaborative Innovation Center for Cancer Medicine, Guangzhou, China; ^7^ Department of General Surgery, Shang Jin Nan Fu Hospital, West China Hospital, Sichuan University, Chengdu, Sichuan, China; ^8^ Department of Neurosurgery, The First Affiliated Hospital of Nanchang University, Nanchang, Jiangxi, China; ^9^ Department of Neurosurgery, Qilu Hospital, Shandong University, Jinan, China; ^10^ Department of Neurosurgery, Tongji hospital, Tongji Medical College, Huazhong University of Science and Technology, Wuhan, China; ^11^ Department of Oral and Maxillofacial Surgery, Tongji Hospital, Tongji Medical College, Huazhong University of Science and Technology, Wuhan, China

**Keywords:** olfactory groove meningioma, communicating, recurrence, prognosis factors

## Abstract

**Objectives:**

Meningioma recurrence remains a significant issue. No study has described the relationship between the clinical features and prognosis of communicating meningioma that primarily originates from the olfactory groove. The aim of the study was to identify prognostic factors of communicating olfactory groove meningiomas that could be stratified according to their risk of recurrence.

**Results:**

A Simpson grade one or two resection was achieved. Complications with cerebrospinal rhinorrhoea occurred in two patients: one required reoperation, and the other was managed successfully with external drainage of lumbar cistern. There were 5 known clinical recurrences within the median follow-up of more than 5 years. The median 5-year recurrence-free survival for patients was 88.4%. Factors such as gender, tumour size, T2 signal and the hyperostotic bone had no significant effect on recurrence-free survival. However, recurrence was activated by oedema range, hyperostosis, dural tail sign and tumor texture (*p* < 0.05). Interestingly, female patients with the disease were younger than males at diagnosis, and the difference was statistically significant (***p =*** 0.013).

**Conclusions:**

Based on these features of communicating olfactory groove meningiomas, different strategies may be adopted for the follow-up and subsequent treatment. Due to the relatively uncommon incidence, more investigations into the clinical behaviour of this entity are crucial.

**Patients and Methods:**

A retrospective study of 43 patients harbouring olfactory groove meningiomas invading the ethmoid or nasal cavity was conducted at three medical centers from 2000 to 2010. The records were reviewed for clinical presentations, imaging studies, surgical observation, histological features and follow-up.

## INTRODUCTION

Meningiomas are commonly considered to grow slowly, and cured by conventional surgery [1]. However, recurrence is often unavoidable due to incomplete tumour resection or subtotal resection [2]. The clinical behaviour of meningiomas originating from olfactory groove has been documented, but very little is known regarding the prognostic factors of olfactory groove meningiomas (OGMs) with extension into the ethmoid sinuses or nasal cavity [3]. The majority of the disease relapses occur among World Health Organization (WHO) grade I patients [4]. Preoperatively failing to understand the clinical behaviour of these tumours can cause inadequate attention for treatment and follow-up. Based on this information, there is an urgent need to identifying reliable prognosis factors of communicating OGM to adopt different treatments and an efficient follow-up strategy for individual patients.

## RESULTS

No patient died of surgery. All patients received clinical examination and MRI assessments. Total resection of tumor by the three neurosurgeons was achieved in the investigated patients. Follow-up procedures were put into effect in accordance with a standard clinical protocol that consisted of MRI performed 3 months after surgery in the first year and every one or two years thereafter. Additional MRI studies were performed whenever clinical signs and/or symptoms were noted and/or a relapse was suspected. At the time of study closing, five patients had recurrent tumours. Histologically, the majority of tumours were the meningothelial type (WHO Tumor classification criteria, 2007). Significant PTBE was associated with a longer hospital stay, difficulty of surgical resection, soft tumor texture, dural tail sign and a risk of intracranial hypertension compared with meningiomas with less oedema in our series.

**Figure 1:**
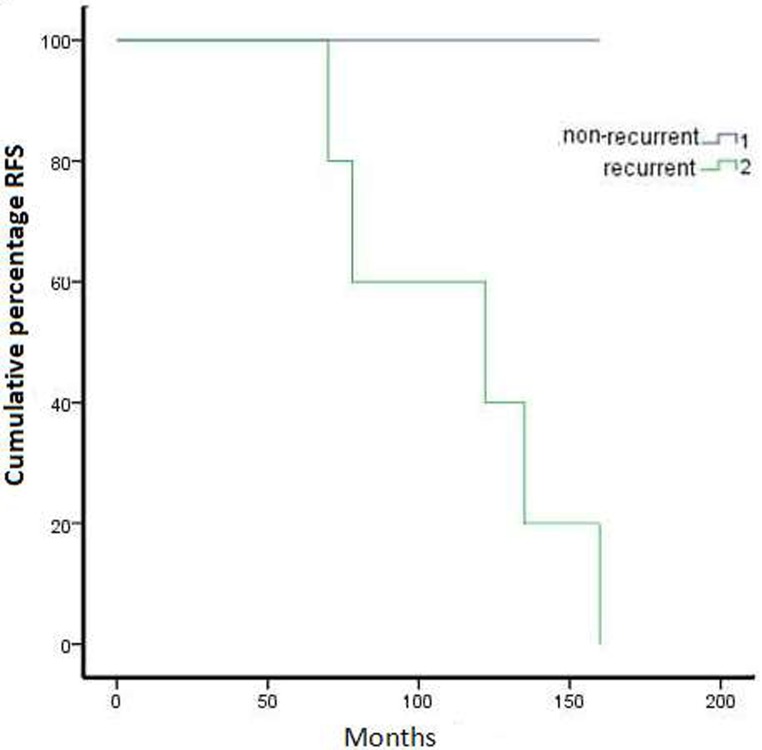
Kaplan–Meier representation of recurrence free survival over five years for all cases with communicating OGM

**Figure 2:**
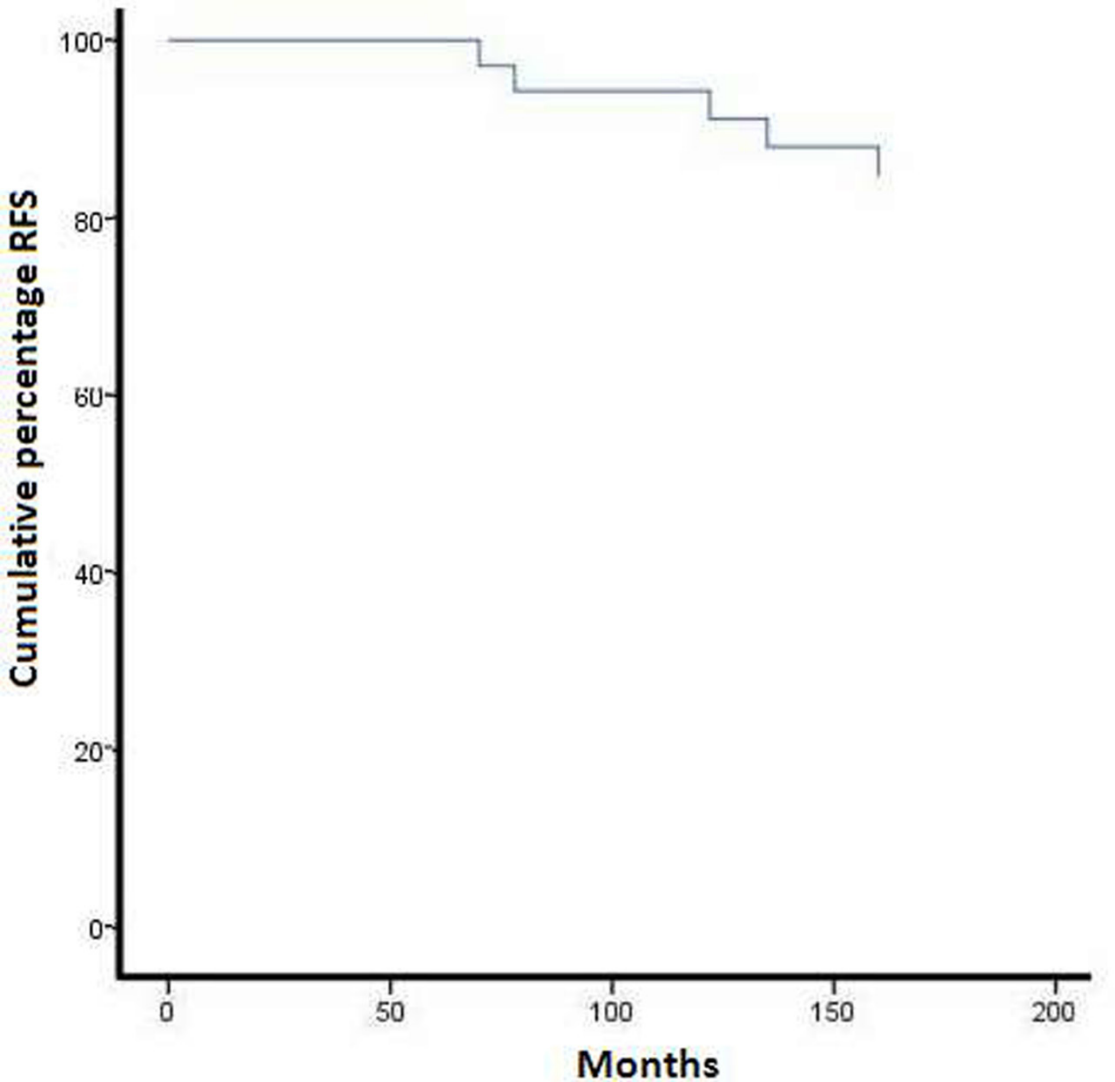
Survival in recurrent and non-recurrent cases

In a detailed analysis of the factors increasing communicating OGM recurrence, five clinical features of the disease exhibited an adverse impact on participants’ RFS, namely younger age based on gender (*P* = 0.026) (Table 1), presence of brain oedema range (bandwidth) ≥20 mm (*P* = .023), soft tumor texture (*p* = 0.037), hyperostosis (*P* = 0.037) and dural tail sign (*p* = 0.019) (Table 2). These factors, including gender (*P* = 0.129), size (*P* = 0.671), T2 signal (*P* = 0.671) and bone hyperostosis (*P* = 0.598), were not able to predict RFS of OGM (Table 2). The overall actuarial disease-free survival for all patients in our series was 88.4% (Figure 1). Five patients developed local tumour relapse (Figure 2).

**Table 1:** Female patients are younger than males at communicating OGM diagnosis

Age/Gender	Female	Male	*P* value
45	10	3	0.026
>45	12	18	

**Table 2:** Clinical and radiographic characteristics of communicating OGM

Variables	Patient distribution	No. of recurrences	*P* value
Gender			
Males	21	2	0.708
Females	22	3	
Oedema range			
≥20 mm	7	3	0.023
<20 mm	36	2	
Tumor size			
≥50 mm	7	1	0.833
<50 mm	36	4	
Tumor texture			
soft	8	3	0.037
hard	35	2	
Dural tail sign			
Yes	12	4	0.019
No	31	1	
Hyperostosis			
Yes	8	3	0.037
No	35	2	

## DISCUSSION

Olfactory groove meningiomas (OGM) arise from the weakest part of the skull base. The cribriform plate has an extension into the ethmoid or nasal cavity, which is an uncommon site of occurrence [5]. Tumours located in this area perhaps vary with respect to clinical characteristics compared with other regions. Forecasting OGM recurrence in a single case remains challenging [1, 2]. This study identified potentially relevant clinical prognostic factors based on long-term follow-up to predict tumour relapse.

In the present study, we introduce OGM with extracranial extension as a clinical entity distinct from meningiomas with a predominant origin from the sphenoid wing, cavernous sinus, convexity, or petrous ridge. We believe that meningiomas that arise predominantly from olfactory groove with extracranial extension have important clinical implications. The most relevant clinical features have been systematically investigated in our surgical series. Males and females each accounted for 50% of the patients. The mean age at presentation was 47 yrs. Of interest, there was a statistically significant difference for the males compared with females in terms of age; females were, on average (44 yrs), younger than their male counterparts (50.2 yrs; *p* = 0.013). We conclude that female patients are younger than their male counterparts at the time of diagnosis. A similar report about the relationship between age and gender in patients with communicating OGM was not searched in the literature. In contrast to our finding, the average age at presentation for women was older than men, which was statistically significant, but the authors did not explain this difference [6]. No significant differences were found with regard to patients’ age and sex distribution among patients [7]. No recorded studies confirmed the prognostic value of patient age [8, 9]. Several studies have demonstrated a worse outcome in younger patients [10, 11].

All patients in our series underwent surgical resection via bilateral or unilateral inferior frontal approach craniotomy, with or without assistance by nasal endoscopy. Complete removal of the OGM was attained in 67 to 100% of the examined cases according to different reports [12, 13]. Macroscopically, the tumours were typically infiltrative into the bone of the nasal cavity or sinus. Hyperostosis was frequently macroscopically visible. Recurrence rate data of OGMs are controversial. Several reports have displayed a very low recurrence rate [14, 15]. In contrast, some studies reported a high recurrence rate of up to 41% at the 10-years follow-up in OGMs [13]. The tumour recurrence rate clearly relies on both the extent of tumour resection and the duration of the follow-up period, but these studies did not assess the relationship between tumour recurrence and clinical and pathological features.

Based on our results, 43 OGM cases with distinct prognoses were defined by a combination of clinical features that served as independent prognostic factor. In total, 11.6% of cases were classified with a bad prognosis indicative of a relapse within 5 years after tumour removal. Five high-risk factors involving obvious oedema (≥20 mm), soft tumor texture, hyperostosis and dural tail sign, indicated a high frequency of recurrence. Some OGM cases with complete resection and greater oedema extension were also associated with a worse prognosis in our series (*p <* 0.05). The specific reasons for the association between oedema range and bad prognosis require further investigation. Our patients all exhibited peritumoural brain oedema (PTBE) on preoperative imaging, which is consistent with previous documents [16]. Oedema was associated with a shorter course of neurological symptoms [17]. Significant PTBE was associated with a longer hospital stay, difficulty of surgical resection, and a risk of intracranial hypertension compared with meningiomas with less oedema in our series. In our view, the easier the surgical plane is to detect, the easier the resection will be. When the arachnoid membrane interface was unclear, the resection was equally arduous. We noticed that patients without or less oedema were inclined to be those in whom the neurosurgeon could discern an explicit plane between the brain tissue and the tumour. This type of interface is frequently associated with PTBE and a disrupted arachnoid layer [18–20], which may explain why meningioma recurs, even following complete removal. Several causative factors for PTBE have been presented to date, including tumour location, size, histological differentiation, sex hormone receptors, venous stasis, vascularity, secretory activity, microcortical invasion, and macrophage infiltrates [21, 22]. However, the relationship between PFS and oedema was not investigated in patients with communicating OGM patients.

To the best of our knowledge, this is the first series of communicating OGM cases reported to date in which clinical traits were systematically investigated with the recurrence of the disease. Oedema range, hyperostosis, soft tumor texture, dural tail sign, and younger female patients with communicating OGM emerged as the relevant prognostic factors. Consequently, these 5 prognostic factors mentioned above were retained at the expense of a decreased frequency of cases. Altogether, these data indicate that the classification superinduces valuable prognostic information to that of the WHO grade alone and result in a more refined risk classification of communicating OGM.

No significant correlation was discovered among tumour sizes, simple gender, T2 signal and recurrence. In our surgical series, the overall tumour recurrence rate was 11.6% during a median follow-up period of 91.8 months. The 5-year locoregional disease control rate in the present series was 88.4%, and the uncovered factors that were independently associated with locoregional recurrence included the involvement of oedema, hyperostosis and dural tail sign. Soft tumor texture equally reduced the RFS. A clustering of cases with bone hyperostosis is expected to be associated with relapse [22], and this finding was supported by our data.

In addition, these tumours clinically behave different from other positions of meningiomas given that grow rather large prior to presentation. By virtue of their subfrontal location, these patients present with nonspecific symptoms. In the light of their large size at diagnosis, it is not surprising that the morbidity of resecting these tumours is not trivial, with many of patients exhibiting obvious postoperative sequelae. We report the clinical characteristics of a group of patients presenting with OGM. Given the uncommon nature of these lesions, 43 consecutive patients were identified from three medical centers. Thus, the data definitively demonstrate a clinical course for these lesions that is truly different from other well-known meningioma classes (ie, cavernous sinus, sphenoid wing, etc). More investigations into the clinical behaviour of this entity, such as karyotype, are necessary before we can make meaningful conclusions about the clinical behaviour of these lesions [23].

## MATERIALS AND METHODS

A total of 43 cases were retrospectively reviewed from three medical centers between 2000 and 2010. Data for these patients, including demographics, gender, radiographic material, pathology data, perioperative findings and postoperative evidence of recurrence on magnetic resonance imaging (MRI), were gathered by review of medical records. The most common presenting symptoms were olfactory impairment (16 patients), cognitive decline (11 patients), memory loss (9 patients), apathia (4 patients) and headache (3 patients). Onset of these symptoms is gradual, and they may not be noted early. These cases comprised 21 males and 22 females, aged 36 to 67 years (mean, 47.0 yrs).

**Figure 3:**
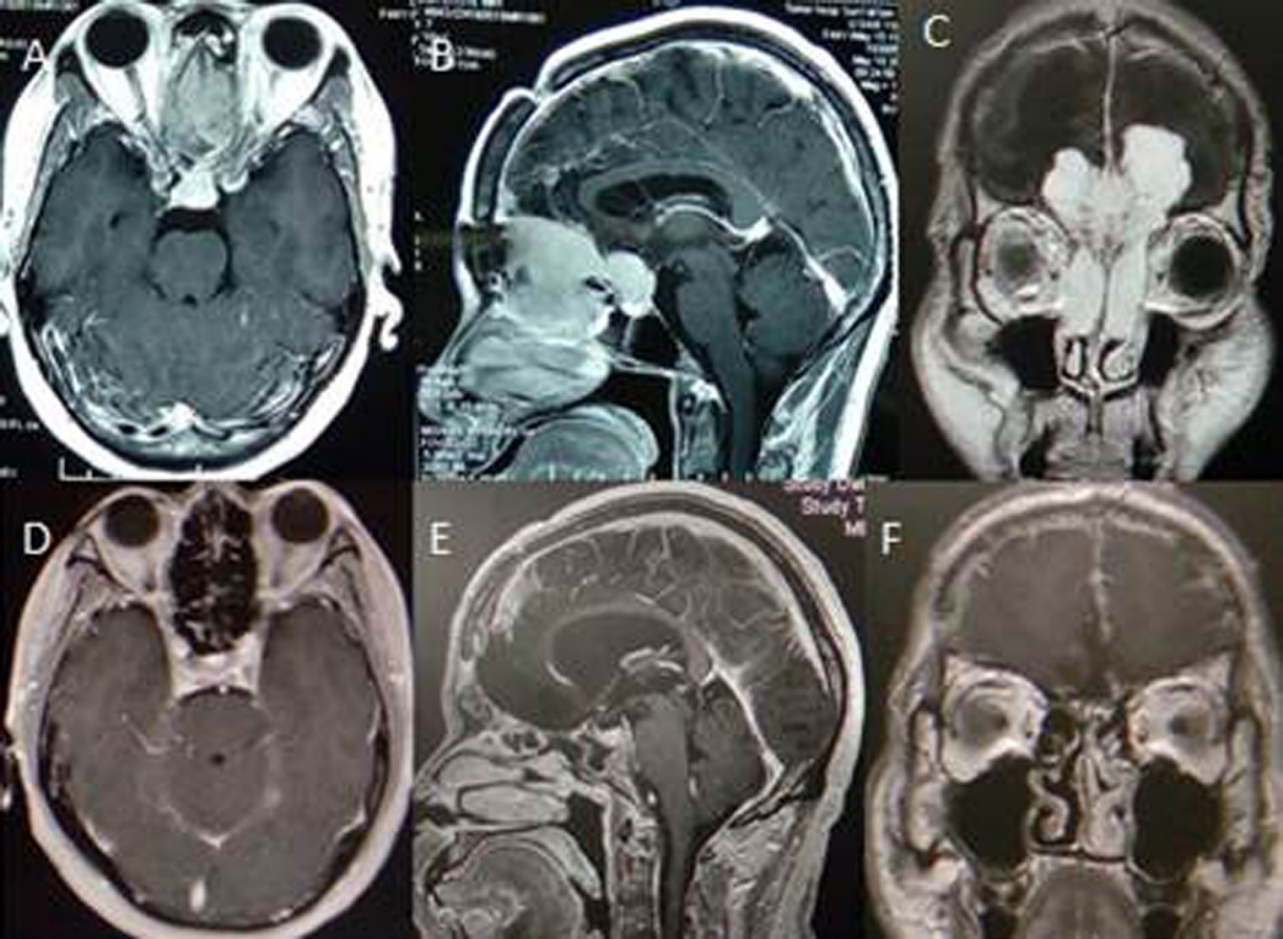
Cranial MR image displaying olfactory groove tumor with nasal cavity extension and follow-up with no recurrence

We reviewed computed tomography (CT) and MR images to identify patients with tumours meeting our definition of OGM with extracranial invasion like (Figure 3A–3C). Tumours ranged from 4.0 to 8.0 cm in diameter (mean, 5.5 cm). The tumours eroded the mucous membrane of the sinuses and extended into the sinal cavity. Age, gender, size, oedema, T2 signal, bone hyperostosis and destruction were analysed in statistics. No adjuvant therapy was administered, with the exception of radiotherapy in two patients. Tumour histological diagnosis coincided with the WHO I criteria, with the exception of one atypical case. The classification of tumour pathology was diversified. All patients received follow-up with clinical examination and MRI studies three months and one year after surgery like Figure 3D–3F. Thereafter, patients were re-examined regularly at one-or two-year intervals based on each follow-up result. The duration of follow-up ranged from 66 to 159 months (median, 91.8 months).

### Surgical findings

A bifrontal/unilateral subfrontal craniotomy with/without endoscopic endonasal assistance was performed, providing wide access to the anterior fossa with minimal frontal lobe retraction while still enabling total tumour removal with a low morbidity rate and no mortality. As shown by preoperative MRI and confirmed intraoperatively, dural thickness around the base of tumour was disclosed in 16 cases; skull base hyperostosis and bone destruction at the tumour attachment area were observed in four and three cases, respectively. Bilateral extension of the tumour into the ethmoidal cells was observed on preoperative coronal and sagittal MRI in 22 cases, and unilateral extension was observed in 21 cases during surgery. Almost all lesions, regardless of the type, demonstrated an infiltrative growth pattern, frequently invading adjacent tissue.

### Pathologic features

Calcifications and fragments of bone were macroscopically visible. Histological diagnosis was performed according to the WHO criteria. The pathologic classification of these neoplasms included meningothelial, transitional, fibrous, and psammomatous meningioma. Epithelial membrane antigen was positive in two-thirds of cases, although they were often weakly and focally observed. Rare lesions exhibited focal immunoreactivity with keratin, CD34, and S-100 protein. All cases were positive for proliferating cell nuclear antigen (PCNA) to a variable degree and intensity. Immunoreactivity was noted for progesterone receptors in all patients more frequently than for oestrogen receptors. Reactivity for smooth muscle actin was observed. The table provides a summary of the immunohistochemistry data obtained in our 43 patients (Table 3).

**Table 3:** Immunohistochemical panel of communicating OGM

Antigen/antibody	Positive	Negative
Epithelial membrane antigen (EMA)	32	11
Vimentin	21	22
Smooth muscle actin (SMA)	16	27
Progesterone receptor	40	3
Estrogen receptor	0	43
Epidermal growth factor receptor (EGFR)	12	31

### Statistical analysis

To establish the statistical significance of differences observed between two variables, the chi-square test was used for qualitative variables. A *P*-value was calculated for each comparison using two-tailed analysis, with significance assumed at the 0.05 level. Recurrence-free survival (RFS) curves were plotted according to the method of Kaplan–Meier method.

## Abbreviations

OGM: Olfactory groove meningioma
WHO: World Health Organization
RFS: Recurrence-free survival
CT: Computed tomography
MRI: Magnetic resonance imaging
PTBE: Peritumoral brain edema
